# NAVKIDS^2^ trial: a multi-centre, waitlisted randomised controlled trial of a patient navigator intervention in children with chronic kidney disease — statistical analysis plan and update to the protocol

**DOI:** 10.1186/s13063-022-06783-y

**Published:** 2022-09-30

**Authors:** Anita van Zwieten, Elizabeth G. Ryan, Patrina Caldwell, Kirsten Howard, Allison Tong, Jonathan C. Craig, Stephen I. Alexander, Martin Howell, Armando Teixeira-Pinto, Carmel M. Hawley, Shilpanjali Jesudason, Amanda Walker, Fiona Mackie, Sean E. Kennedy, Steven McTaggart, Hugh J. McCarthy, Simon A. Carter, Siah Kim, Reginald Woodleigh, Anna Francis, Alistair R. Mallard, Amélie Bernier-Jean, David W. Johnson, Deirdre Hahn, Donna Reidlinger, Elaine Pascoe, Julie Varghese, Charani Kiriwandeniya, Liza Vergara, Nicholas Larkins, Luke Macauley, Michelle Irving, Rabia Khalid, Chandana Guha, Germaine Wong

**Affiliations:** 1 Centre for Kidney Research at The Children’s Hospital at Westmead, Westmead, New South Wales Australia; 2 Sydney School of Public Health, University of Sydney, Sydney, New South Wales Australia; 3 Australasian Kidney Trials Network, University of Queensland, Brisbane, Australia; 4 QCIF Facility for Advanced Bioinformatics, Institute for Molecular Bioscience, The University of Queensland, Brisbane, Australia; 5 Discipline of Child and Adolescent Health, Faculty of Medicine and Health, The University of Sydney, Sydney, New South Wales Australia; 6 College of Medicine and Public Health, Flinders University, Adelaide, South Australia Australia; 7 Department of Nephrology, Princess Alexandra Hospital, Brisbane, Queensland Australia; 8 Translational Research Institute, Brisbane, Australia; 9 Central Northern Adelaide Renal and Transplantation Service (CNARTS), Royal Adelaide Hospital, Adelaide, South Australia Australia; 10 Department of Nephrology, Royal Children’s Hospital, Melbourne, Victoria Australia; 11 Department of Nephrology, Sydney Children’s Hospital, Randwick, New South Wales Australia; 12 School of Clinical Medicine, Faculty of Medicine & Health, University of New South Wales, Sydney, Australia; 13 Children’s Health Queensland Hospital and Health Service, Brisbane, Queensland Australia; 14 Nephrology Department, The Children’s Hospital at Westmead, Westmead, New South Wales Australia; 15 Prostate and Breast Cancer Foundation (CanCare), Hurstville, Australia; 16 School of Human Movement and Nutrition Sciences, University of Queensland, Brisbane, Queensland Australia; 17 Centre de Recherche du CIUSSS du Nord-de-l’Île de Montréal, University of Montréal, Montréal, Canada; 18 Perth Children’s Hospital, Perth, Australia; 19 Kidney Health Australia, Melbourne, Australia; 20 Menzies Centre for Health Policy, The University of Sydney, Camperdown, NSW 2006 Australia; 21 Centre for Evidence and Implementation, Carlton, Victoria Australia; 22 Department of Renal Medicine, Westmead Hospital, Westmead, New South Wales Australia

**Keywords:** Chronic kidney disease, Dialysis, Kidney transplantation, Children, Adolescents, Patient navigator, Socioeconomic disadvantage, Health disparities, Randomised controlled trial

## Abstract

**Background:**

This update summarises key changes made to the protocol since the publication of the original protocol for the NAVKIDS^2^ trial of patient navigators for children with chronic kidney disease (CKD) experiencing social disadvantage and provides the statistical analysis plan (SAP) which has not previously been published.

**Methods/design:**

The original protocol was published in *BMC Nephrology* (10.1186/s12882-019-1325-y) prior to the commencement of trial recruitment. During the course of the trial, some key methodological changes needed to be made including changes to eligibility criteria (addition of patients with CKD stages 1–2, broadening of financial status eligibility criterion, addition of patients living in rural/remote areas, modification of age eligibility to 0–16 years, addition of limits related to the language spoken by family, guidance regarding families with multiple eligible children), changes to sites, reduction of sample size, addition of virtual options for consent and study procedures in response to the COVID-19 pandemic, removal of staggered recruitment across sites, addition of outcomes, and changes to the timing and number of assessments. This update summarises the changes made and their rationale and provides the detailed plan for statistical analysis of the trial. These changes have been finalised prior to the completion of study follow-up and the commencement of data analysis.

**Trial registration:**

Australian New Zealand Clinical Trials Registry (ANZCTR) ACTRN12618001152213. Prospectively registered on 12 July 2018

**Supplementary Information:**

The online version contains supplementary material available at 10.1186/s13063-022-06783-y.

## Administrative information

**Table:** 

Title	NAVKIDS^2^ trial: a multi-centre, waitlisted randomised controlled trial of a patient navigator intervention in children with chronic kidney disease – statistical analysis plan and update to the protocol
Trial registration	Prospectively registered (12/07/2018) on the Australian New Zealand Clinical Trials Registry (ANZCTR) (ACTRN12618001152213).
Protocol version	Update to previously published protocol [1]. This update reflects study protocol v9 dated 6/12/21.
Funding	This work is funded by a National Health and Medical Research Council (NHMRC) Medical Research Future Funds grant (APP 1170021). AvZ, SC and CG have received funding from NHMRC postgraduate scholarships (AvZ APP1115259, SC APP1168994, CG APP2014258). GW and AT are recipients of NHMRC Career Development Fellowships (GW APP1147657 and AT APP1106716). MH and ATP receive support from a NHMRC Program Grant (APP1092957). DJ is the recipient of a NHMRC Leadership Investigator Grant (APP1194485). The funding organizations will have no involvement in the design and conduct of the study; collection, management, analysis, and interpretation of the data; preparation, review or approval of the manuscript; or the decision to submit the manuscript for publication.
Author details	Anita van Zwieten*^1,2^, Elizabeth G. Ryan*^3,4^, Patrina Caldwell^1,5^ , Kirsten Howard^2^, Allison Tong^1,2^, Jonathan C Craig^6^, Stephen I Alexander^1,2^, Martin Howell^1,2^, Armando Teixeira-Pinto^1,2^, Carmel M Hawley^3,7,8^, Shilpanjali Jesudason^9^, Amanda Walker^10^, Fiona Mackie^11^, Sean E Kennedy^11,12^, Steven McTaggart^13^, Hugh J McCarthy^1,5,11,14^, Simon A Carter^1,2,10^, Siah Kim^1,2^, Reginald Woodleigh^15^, Anna Francis^13^, Alistair R Mallard^16^, Amélie Bernier-Jean^17^, David W Johnson^3,7,8^, Deirdre Hahn^1,14^, Donna Reidlinger^3^, Elaine Pascoe^3^, Julie Varghese^3^, Charani Kiriwandeniya^3^, Liza Vergara^3^, Nicholas Larkins^18^, Luke Macauley^19^, Michelle Irving^20, 21^, Rabia Khalid^1,2^, Chandana Guha^1,2^, Germaine Wong^1,2,22^ *These authors contributed equally ^1^Centre for Kidney Research at The Children’s Hospital at Westmead, Westmead, New South Wales. ^2^Sydney School of Public Health, University of Sydney, Sydney, New South Wales. ^3^Australasian Kidney Trials Network, University of Queensland, Brisbane, Australia. ^4^QCIF Facility for Advanced Bioinformatics, Institute for Molecular Bioscience, The University of Queensland, Brisbane, Australia. ^5^Discipline of Child and Adolescent Health, Faculty of Medicine and Health, The University of Sydney, Sydney, New South Wales. ^6^College of Medicine and Public Health, Flinders University, Adelaide, South Australia. ^7^Department of Nephrology, Princess Alexandra Hospital, Brisbane, Queensland. ^8^Translational Research Institute, Brisbane, Australia. ^9^Central Northern Adelaide Renal and Transplantation Service (CNARTS); Royal Adelaide Hospital, South Australia. ^10^Department of Nephrology, Royal Children’s Hospital, Melbourne, Victoria. ^11^Department of Nephrology, Sydney Children’s Hospital, Randwick, New South Wales. ^12^School of Clinical Medicine, Faculty of Medicine & Health, University of New South Wales, Sydney, Australia. ^13^Children’s Health Queensland Hospital and Health Service, Brisbane, Queensland. ^14^Nephrology Department, The Children’s Hospital at Westmead, Westmead, New South Wales. ^15^Prostate and Breast Cancer Foundation (CanCare), Australia. ^16^School of Human Movement and Nutrition Sciences, University of Queensland, Brisbane, Queensland. ^17^Centre de Recherche du CIUSSS du Nord-de-l'Île de Montréal, University of Montréal, Montréal, Canada. ^18^Perth Children’s Hospital, Perth, Australia. ^19^Kidney Health Australia, Melbourne, Australia. ^20^Menzies Centre for Health Policy, The University of Sydney, Camperdown, NSW 2006, Australia. ^21^Centre for Evidence and Implementation, Carlton, Victoria, Australia. ^22^Department of Renal Medicine, Westmead Hospital, Westmead, New South Wales, Australia
Name and contact information for the trial sponsor	The University of Queensland acting through Australasian Kidney Trials Network (AKTN). Email: aktn@uq.edu.au
Role of sponsor	The sponsor is the coordinating center for the trial and is involved in overall study activities including study design, collection, management, analysis and interpretation of data, writing of the report, and decision to submit the report for publication.

## Update

This is an update to the protocol for the NAVKIDS^2^ trial, which was originally published in *BMC Nephrology* [1]. The NAVKIDS^2^ trial is a multi-centre, waitlisted randomised controlled trial that assesses the health benefits and costs of a patient navigator programme in children with chronic kidney disease (CKD) stages 1–5, on dialysis (CKD-D) and with kidney transplants (CKD-T), who are from low socioeconomic status (SES) backgrounds and/or living in rural/remote areas. The key research question is whether a patient navigator programme improves self-rated health at 6 months post-randomisation compared to standard care in children with CKD (1–5), CKD-D and CKD-T who are from low SES backgrounds and/or living in rural/remote areas. Throughout the course of the study, some changes needed to be made to the study protocol. The key changes made and rationale for the changes are outlined in Additional file 1. As detailed in Additional file 1, the changes relate to the following areas: objectives and outcomes, eligibility criteria, study sites and recruitment, consent, randomisation, intervention, follow-up and data collection, trial oversight and compliance, and statistical analyses. A few of the key changes are additionally described in text here, and the full revised protocol can be requested from the corresponding authors via email.

In regard to the eligibility criteria, we have made a number of changes in order to ensure that the intervention was targeted towards a suitable population with relevant needs that could be met by patient navigation and to ensure the feasibility of recruitment. We expanded the CKD criterion to include children with CKD stages 1–2 (in addition to stages 3–5, dialysis and transplant), because socioeconomic inequalities in health may be even greater among children and adolescents with earlier stages of CKD than those on kidney replacement therapy (KRT) [2, 3], and this group may benefit from early intervention. Originally, the trial was limited to children experiencing socioeconomic disadvantage, but we have now expanded the eligibility to additionally include children living in rural or remote areas of Australia. Living in rural/remote areas is an established social determinant of health and our research has indicated that children with kidney failure living in remote/regional Australia are less likely to access optimal care, which suggests that this group may benefit from an intervention to improve health care access [4]. We have also changed the age eligibility criterion to 0–16 years (from 3–17 years), reducing the lower limit as children are commonly diagnosed during early childhood and families may benefit from early intervention, and reducing the upper limit as patients aged 17–18 years are often in the process of transitioning to the adult hospital and have specific needs in this stage. We also expanded the financial status eligibility criterion, in order to broaden the eligible population and align with our definitions of disadvantage in previous related work [2, 3]. We have also provided guidance related to recruitment for families with more than one eligible child, and for feasibility reasons, we have added a requirement that caregiver(s) speak English or have a family member who can speak English.

There were also a number of changes made due to the COVID-19 pandemic and associated stay-at-home measures in Australia during this period. These included the addition of verbal consent processes, addition of virtual study procedures (including virtual navigation and changing blood collection and physical examination to non-mandatory outcomes so that data collection could be completed virtually), reduction of follow-up duration and reduction in the target sample size from 210 to 150–168 participants. The sample size and follow-up duration were reduced as recruitment started late due to the lockdown period during the COVID-19 pandemic. In relation to sample size specifically, the dropout was expected to be low; therefore, the study power was preserved at approximately 80%. These changes enabled us to ensure successful execution of trial recruitment and follow-up processes within the grant timeline and budget. The addition of verbal consent and virtual study procedures enabled us to protect the safety of participants and study staff.

We have added clarification that safety monitoring will be undertaken by a Safety Monitoring Committee (SMC) as this is the most appropriate type of monitoring for this trial, and have added extensive details concerning trial oversight and compliance in accordance with recommendations of the Standard Protocol Items: Recommendations for Interventional Trials (SPIRIT) checklist [5]. These include details of the role of the sponsor, Trial Steering Committee and Trial Management Committee, quality assurance and auditing, ancillary and post-trial care, data handling and confidentiality, data sharing and dissemination of results.

We have also made a number of updates to the plan for statistical analysis in consultation with the trial statisticians and present the detailed statistical analysis plan (SAP) for the study in Additional file 2, which has not previously been published, alongside a completed SAP checklist in Additional file 3 in accordance with the Guidelines for the Content of Statistical Analysis Plans in Clinical Trials [6].

## Trial status

Protocol version 9 date 6/12/2021

Recruitment start date: 17/07/2020

Recruitment end date: 12/10/2021

## Supplementary Information

**Additional file 1.** Summary of key changes to protocol including rationale

**Additional file 2.** NAVKIDS^2^ Statistical Analysis Plan (SAP)

**Additional file 3.** SAP checklist in accordance with the Guidelines for the Content of Statistical Analysis Plans in Clinical Trials

## Data Availability

Not applicable.

## Abbreviations

ANZCTR: Australian New Zealand Clinical Trials Registry
CKD: Chronic kidney disease
CKD-D: Chronic kidney disease on dialysis
CKD-T: Chronic kidney disease with a kidney transplant
COVID-19: Coronavirus disease 2019
KRT: Kidney replacement therapy
SAP: Statistical analysis plan
SES: Socioeconomic status
SMC: Safety Monitoring Committee
SPIRIT: Standard Protocol Items: Recommendations for Interventional Trials
