# Supplementary material for: NAVKIDS2 trial: a multi-centre, waitlisted randomised controlled trial of a patient navigator intervention in children with chronic kidney disease — statistical analysis plan and update to the protocol
Source: Trials. 2022 Sep 30;23:824. doi: 10.1186/s13063-022-06783-y (PMC9522454; doi:10.1186/s13063-022-06783-y)
Supplement: Supplementary file 1 — Additional file 1. Summary of key changes to protocol including rationale [file 13063_2022_6783_MOESM1_ESM.docx]

**Additional File 1. Summary of key changes to protocol including rationale.**

| **Area** | **Change made** | **Rationale for change** |
| --- | --- | --- |
| **General** | **Addition and removal of authors** | We have added authors who are members of the Trial Steering Committee including site staff, researchers and consumer representatives, and central coordinating staff. We have removed one author who is no longer involved in the trial due to changes in study sites (as detailed below). |
|  | **Changes to citations/references** | Update of references reflecting publication and correction of manuscripts, addition of references supporting new information, removal of references supporting deleted information. |
| **Objectives and outcomes** | **Edit of key research question to focus on the primary outcome (rather than being broadly worded across outcomes).** | This was edited to ensure a focus on the primary outcome of the study. |
|  | **Clarification of objectives and outcomes wording: secondary objectives and outcomes will not all be examined as means, outcomes reframed to remove reference to comparisons.** | Previous wording for secondary objectives and outcomes implied all outcomes would be compared using means - this has been updated in consultation with our study statisticians. Comparisons removed from outcomes as this is addressed in the objectives. |
|  | **Qualitative study moved to secondary objectives (previously tertiary). Progression of kidney dysfunction and other biomarkers moved to exploratory outcomes (previously secondary).** | The qualitative component of the study has been moved to the secondary objectives as the qualitative and quantitative findings will be presented together in the main results paper to support interpretation of each other. Biomarkers have been moved to exploratory outcomes as they will not be presented in the main paper. |
|  | **Divided secondary outcomes into ‘key’ secondary outcomes vs. others. Clarification that CKD-related outcomes are other secondary outcomes.** | This distinction was added because only the key secondary outcomes will be presented in the main results paper. Clarification was added about CKD-related outcomes because these were mentioned in outcome measurement but not in the listing of secondary outcomes. |
| **Eligibility Criteria** | **Expansion of eligibility to include children with chronic kidney disease (CKD) stages 1-2 (in addition to CKD stages 3-5, dialysis and transplant).** | Our research has indicated that socioeconomic inequalities in health may be greater among children and adolescents with earlier stages of CKD than those on kidney replacement therapy (KRT) (1, 2). Further, our qualitative work has indicated that adult patients with CKD endorsed the need for patient navigation in early stages of disease (3). We believe that children and families with CKD stages 1-2 may benefit from a patient navigator guiding them through the health care system during the early stages of the disease. |
|  | **Expansion of eligibility to include children living in rural or remote areas classified as RA2-RA5 (in addition to children experiencing low SES).** | Our research has indicated that children with kidney failure living in remote/regional Australia are less likely to access optimal care including pre-emptive living donor kidney transplantation and are therefore a disadvantaged group in terms of health care access (4). Patient navigators may be able to increase the accessibility of community and healthcare facilities for these patients and their caregivers. |
|  | **Change of age eligibility to 0-16 years (from 3-17 years).** | Lower age limit reduced to 0 years as there are many children who are diagnosed with CKD before the age of 3 years, and having the navigator program at an early stage of diagnosis may be helpful to patients and their families. Upper age limit reduced to 16 years as many children move to the adult hospital when they turn 18 years of age, and they may not wish to return for study visits at the children’s hospital, which may increase the number of withdrawals. Further, the needs of 17-18 year-old patients who are transitioning to the adult hospital are likely to differ from those of younger patients and are likely to require a program with a focus on transition. |
|  | **Expansion of financial status eligibility to include families with self-perceived financial status of “just getting along” (in addition to “poor” and “very poor”). Clarification that SES criteria are self-reported.** | We added this group to align with definitions of socioeconomic disadvantage in our previous related work [1] and broaden the eligible population. We have clarified that SES criteria are self-reported to provide further clarity about our included population. |
|  | **Addition of inclusion criterion that only one sibling from each family can be included in the study.** | The inclusion of siblings in NAVKIDS^2^ was not explicitly discussed or accounted for in the trial design at the outset. Individually randomising siblings into the trial is a problem because there is a 50% chance they will be allocated to different treatment groups. This would result in treatment ‘contamination‘ because the sibling who accesses the navigator first will ‘contaminate’ the remaining sibling who is wait-listed to receive the navigator intervention. Treatment contamination would likely reduce the size of the treatment effect. If only one sibling is included:   - Treatment contamination can be avoided. - Current sample size calculation will remain valid. - If the navigator program is effective, other siblings would still receive indirect exposure to the intervention.   The decision about which sibling to enrol in the study will be made by parents/caregivers as they are likely to have the best understanding of their child’s health and medical/service requirements. |
|  | **Addition of inclusion criterion that caregiver(s) speak English or caregiver(s) speaks a little English but has a family member who can speak English.** | Participants who do not speak English and do not have caregivers/family members who speak English will be excluded for feasibility reasons (including costs of repeated translation, feasibility of recruiting multiple patient navigators fluent in the varied languages of the study population). To maximise generalisability and equity of access, families in which caregivers speak only a little English but can be assisted by English-speaking family members will be included. This level of communication will be sufficient for the navigator in assisting participants with day-to-day navigation activities. These families will have access to a phone interpreter service during consent and study visits for data collection. The key study information sheet will be translated to some of the most commonly used languages other than English. |
|  | **Rewording of consent eligibility criterion to families being included if able to provide consent (from families being excluded if unable/unwilling to consent).** | This was moved to the inclusion criteria as study staff should confirm that participants are willing to consent to the study before proceeding to checking exclusion criteria. |
| **Study sites and recruitment** | **Change of fifth recruitment site to Perth Children’s Hospital (from the Women’s and Children’s Hospital Adelaide).** | This change was made because recruitment was more feasible at this site. |
|  | **Change of target sample size to 150-168 participants (from 210), and recruitment per site to up to 42 participants per site (from 42 per site).** | Study recruitment started late due to COVID-19 restrictions, leading to reduced recruitment time. Thus, the study recruitment target was changed from 210 to 150-168 as the dropout rate is expected to be low, and this revised target was feasible within the grant timeline and budget. The number of participants per site was amended to reflect the reduced overall sample size and account for variability in start dates and rates of recruitment across sites. |
|  | **Recruitment changed to enable concurrent recruitment across sites (rather than being staggered in structured waves, with each site commencing at the start of a school term), hence removal of the term “staggered” from study design.** | This change was made for feasibility reasons in terms of the practicality for site recruitment and study conduct. |
|  | **Change of minimum sample size for qualitative interviews to min. 20 in each group - children/families and study staff (from 30 in each group), with interviews to cease when data saturation is reached.** | We amended this in consultation with our qualitative team members. The team has clarified that qualitative interview studies typically require a minimum sample size of 20 to reach data saturation. Interviews will be undertaken with a minimum of 20 in each group until no new topics/information are revealed. |
| **Consent** | **Addition of the option of verbal informed consent (and verbal assent for children as appropriate) where written consent/assent is not practicable.** | The option of verbal informed consent was added due to practical barriers to written consent during the COVID-19 pandemic and associated stay-at-home measures. Where practicable, written consent will also be obtained later from those who consented verbally. |
|  | **Clarification that consent will be completed by site investigator’s delegate (previously specified trial coordinator or research assistant).** | This was changed so that the site investigator for the study could delegate the responsibility to the study personnel who were trained in the study and considered appropriate to obtain consent for the study at the site. |
| **Randomisation** | **Randomisation will be completed in the REDCap database management system by site personnel (rather than centrally by AKTN), so will not be separated from recruitment. General information about the randomisation process and REDCap has also been added to the protocol.** | This was changed as it was more practical for site personnel to complete randomisation. Allocation concealment will still be maintained because the randomisation is done in the database management system. |
| **Intervention** | **Medical records were removed from the networks aspect of the navigator matrix, making it a four-by-four matrix (from four-by-five).** | This was removed from the navigator matrix because navigators were not able to edit medical records. |
|  | **Clarification added that navigators could work with patients face-to-face or virtually, as appropriate.** | This was added to clarify that navigators worked in the modality suited to the family and situation, including face-to-face or virtual modalities. |
| **Follow-up and data collection** | **Reduction of follow-up duration to 12-months post-randomisation for both groups, with final follow-up to be 6-months post-intervention for intervention arm and immediately post-intervention for waitlist arm (from 12-months post-intervention for both groups). Update of trial schema (Figure 1) and reduction of timepoints for various outcome assessments to align with this change (Figures 2 and 3). We have also updated the schedules for assessment from the original tables (Tables 1 and 2) to be figures using the SPIRIT schedule template (now referred to as Figures 2 and 3).** | Study recruitment started late due to COVID-19 restrictions, leading to reduced recruitment time and concerns about the study budget. The follow-up duration was reduced to enable trial completion within budget. |
|  | **Reduction of the number of qualitative interviews to maximum of two, occurring before and after the intervention in both groups (previously 4 interviews per group).** | The number of interviews was reduced because conducting interviews during the intervention period may contaminate the treatment effect. For the purposes of process evaluation, one interview will be conducted before participants commence the intervention followed up with a post intervention interview. The interviews will be conducted until data saturation is reached for each time point (pre-intervention and post-intervention), meaning that the number of participants interviewed in each time point may vary depending on when saturation is reached (i.e. some participants may only have 1 interview). |
|  | **Clarification of a +/- 2-week window for study visits in assessment schedules (previously no window stipulated).** | This was added because it is not always practicable to have study visits on the exact scheduled day, given families’ other commitments. To avoid protocol deviations, a visit window was added. |
|  | **Clarification of timeframe for measurement of primary outcome child SRH as 6-months post-randomisation (previously 6-months after completion of the intervention). Clarification that child SRH mentioned in secondary outcomes is child SRH over time.** | The previous wording was ambiguous. Revised wording clarifies that the primary outcome will be measured at 6-months post-randomisation, which is immediately post-intervention for the immediate start group and pre-intervention for the waitlist group. Clarification that the secondary outcome is child SRH over time is to clearly differentiate this from the primary outcome of child SRH at 6-months post-randomisation. |
|  | **Change to consistently refer to visits in terms of months (e.g. 6-months post-randomisation) instead of weeks (e.g. 24 weeks).** | Use of months instead of weeks is to account for visit windows and to enhance clarity. |
|  | **Reduction of the number of timepoints where medication data are collected to 2 for immediate group and 3 for waitlist group (previously assessed at every visit from randomisation onwards).** | To reduce the burden of information collection for the study team and participants. Where practicable, detailed information on medication use will be obtained from MBS/PBS linkage at the completion of the study. |
|  | **Addition of biomarkers – calcium, phosphate and intact parathyroid hormone.** | These are surrogate markers of metabolic bone health in children with chronic kidney disease, which is an important outcome recognised by patients and caregivers. |
|  | **Addition of patient navigator activity reports as an outcome under the process evaluation. Addition of time spent with navigator to qualitative interview topics.** | This will assist in evaluating patient navigator time requirements. |
|  | **Blood collection and physical examination changed to non-mandatory outcomes.** | This change was made because of caregiver concerns about additional blood tests, and because it was not feasible to require face-to-face physical examination and non-routine blood collection during the COVID-19 pandemic and associated stay-at-home measures. |
|  | **Clarification of outcome measurement for new age group of 0-2 years – all measurements to be undertaken except HUI quality of life. Clarification that a sensitivity analysis will be conducted for HUI outcomes, excluding children aged 3-4 years. Clarification that HUI will preferably be completed by child.** | Rationale for age change is given above. HUI measurement will not occur for children aged 0-2 years as some questions are difficult to answer for very young children. A sensitivity analysis excluding children aged 3-4 years will be undertaken because although the HUI has been used for young children in previous studies, it has only been validated for children aged 5 and above (5). |
|  | **Change of data linkage timepoint to 12-months post-randomisation for both groups (from 12-months post-treatment in each group). Clarification of when linkage will occur.** | Reflects reduction in follow-up duration for study described above, and harmonises actual time of linkage across both groups. |
|  | **Hospitalisation will be measured using medical records (in addition to caregiver reports). Clarification that healthcare costs will be estimated using urgency related groups (URG) as well as diagnosis-related groups (DRG) and Medicare unit costs.** | As caregivers may not recall hospital admission details at the next study visit, this information will also be collected from hospital medical records. Urgency-related groups (URG) were added as these are the relevant code for emergency department data. |
|  | **Clarification that blinding will be possible only for data analysts, not for outcome assessors.** | This is because outcomes are being collected by site research personnel and it is not practical to keep these staff blinded to intervention status. |
| **Trial oversight and compliance** | **Clarification that safety monitoring will be undertaken by a Safety Monitoring Committee (SMC) rather than a Data Safety Monitoring Board (DSMB) and the SMC will monitor safety of patient navigators as well as children and families. Clarification of independence of SMC and description of expertise for SMC members.** | In line with National Health and Medical Research Council (NHMRC) guidance on ensuring that safety monitoring is appropriately tailored to each trial (6), monitoring will be undertaken by a SMC instead of DSMB because the primary role needed is that of monitoring safety for patients, families and patient navigators. Data integrity is able to be monitored by the Trial Steering Committee. Monitoring of patient navigator safety is a critical role for the SMC in this trial, so this has been explicitly clarified in the protocol. We have added clarification of specific types of expertise relevant to the SMC for this trial and that the SMC is independent. |
|  | **Addition of details about sponsor and role of sponsor. Addition of details about Trial Steering Committee (TSC) and Trial Management Committee (TMC). Addition of details about quality assurance and auditing. Addition of details about ancillary and post-trial care.** | Added to provide further details about trial oversight and compliance as recommended by SPIRIT checklist. |
|  | **Addition of details about data handling, security and confidentiality, data sharing and dissemination of results.** | Added to provide further details about compliance as recommended by SPIRIT checklist. |
|  | **Addition of details about procedures for changes to study methodology.** | Added to provide further details about compliance as recommended by SPIRIT checklist. |
| **Statistical analyses** | **Updates to the plan for statistical analysis and provision of the detailed statistical analysis plan (SAP).** | In consultation with the study statisticians, updates have been made to finalise the plan for statistical analysis. Further details of the plan for analysis are provided in the statistical analysis plan (SAP), which is attached as **Additional File 2**. The SAP follows the Guidelines for the Content of Statistical Analysis Plans in Clinical Trials (7), and a completed checklist is attached in **Additional File 3**. |

**References**

1. Didsbury M, van Zwieten A, Chen K, James LJ, Francis A, Kim S, et al. The association between socioeconomic disadvantage and parent-rated health in children and adolescents with chronic kidney disease—the Kids with CKD (KCAD) study. Pediatr Nephrol. 2019;34(7):1237-45.

2. Didsbury M, van Zwieten A, Chen K, James LJ, Francis A, Kim S, et al. Correction to: The association between socioeconomic disadvantage and parent-rated health in children and adolescents with chronic kidney disease—the Kids with CKD (KCAD) study. Pediatr Nephrol. 2022;37:693-6.

3. Guha C, Lopez-Vargas P, Ju A, Gutman T, Scholes-Robertson NJ, Baumgart A, et al. Patient needs and priorities for patient navigator programmes in chronic kidney disease: a workshop report. BMJ Open. 2020;10(11):e040617.

4. Francis A, Didsbury M, Lim WH, Kim S, White S, Craig JC, et al. The impact of socioeconomic status and geographic remoteness on access to pre-emptive kidney transplantation and transplant outcomes among children. Pediatr Nephrol. 2016;31(6):1011-9.

5. Rowen D, Keetharuth AD, Poku E, Wong R, Pennington B, Wailoo A. A Review of the Psychometric Performance of Selected Child and Adolescent Preference-Based Measures Used to Produce Utilities for Child and Adolescent Health. Value in Health. 2021;24(3):443-60.

6. Data Safety Monitoring Boards (DSMBs). Canberra: National Health and Medical Research Council (NHMRC); 2018.

7. Gamble C, Krishan A, Stocken D, Lewis S, Juszczak E, Doré C, et al. Guidelines for the Content of Statistical Analysis Plans in Clinical Trials. Jama. 2017;318(23):2337-43.
