# Supplementary material for: NAVKIDS2 trial: a multi-centre, waitlisted randomised controlled trial of a patient navigator intervention in children with chronic kidney disease — statistical analysis plan and update to the protocol
Source: Trials. 2022 Sep 30;23:824. doi: 10.1186/s13063-022-06783-y (PMC9522454; doi:10.1186/s13063-022-06783-y)
Supplement: Supplementary file 2 — Additional file 2. NAVKIDS2 Statistical Analysis Plan (SAP) [file 13063_2022_6783_MOESM2_ESM.pdf]

STATISTICAL ANALYSIS PLAN

Version: 1.3

Date: 10/05/2022

Protocol Title: NAVKIDS^2 trial: A multicenter, waitlisted, randomised controlled trial of patient navigators in children with chronic kidney disease

Short title: NAVKIDS^2

Protocol date: 06/12/2021

Protocol version: 9.0

Trial Registration: Australian New Zealand Clinical Trials Registry ACTRN 12618001152213

Australasian Kidney Trials Network
Translational Research Institute, 37 Kent Street, Woolloongabba QLD 4102

DOCUMENT HISTORY

Version	Reason(s) for change	Date
1.0	Initial document	28/09/2021
1.1	Updated to reflect protocol v9.0	25/02/2022
1.2	Updated blinding section (section 2.5) to reflect updated protocol paper	02/05/2022
1.3	Updated to add details to comply with SAP checklist	10/05/2022

APPROVALS

Chief Principal Investigator & Chair of Trial Steering Committee

Name, Affiliation	Signature	Date
Prof Germaine Wong<br>Centre for Kidney Research,<br>The Children's Hospital at Westmead, Westmead, Australia	Germaine Wong<br>Digitally signed by Germaine Wong<br>Date: 2022.05.12 11:53:50 +10'00'	12/5/22

Trial Statistician / Statistical Analysis Plan Author

Name, Affiliation	Signature	Date
Dr Elizabeth Ryan<br>Australasian Kidney Trials Network, The University of Queensland, Brisbane, Australia		12/5/22

Senior Statistician

Name, Affiliation	Signature	Date
Prof Armando Teixeira-Pinto<br>Sydney School of Public Health, The University of Sydney, Sydney, Australia		12/5/22

Chair, Executive Operations Secretariat, Australasian Kidney Trials Network

Name, Affiliation	Signature	Date
Prof Carmel Hawley<br>Australasian Kidney Trials Network, The University of Queensland, Brisbane, Australia		12/5/22

DOCUMENT HISTORY

Version	Reason(s) for change	Date
1.0	Initial document	28/09/2021
1.1	Updated to reflect protocol v9.0	25/02/2022
1.2	Updated blinding section (section 2.5) to reflect updated protocol paper	02/05/2022
1.3	Updated to add details to comply with SAP checklist	10/05/2022

APPROVALS

Chief Principal Investigator & Chair of Trial Steering Committee

Name, Affiliation	Signature	Date
Prof Germaine Wong<br>Centre for Kidney Research,<br>The Children's Hospital at Westmead, Westmead, Australia		

Trial Statistician / Statistical Analysis Plan Author

Name, Affiliation	Signature	Date
Dr Elizabeth Ryan<br>Australasian Kidney Trials Network, The University of Queensland, Brisbane, Australia	Elizabeth Ryan	12/05/2022

Senior Statistician

Name, Affiliation	Signature	Date
Prof Armando Teixeira-Pinto<br>Sydney School of Public Health, The University of Sydney, Sydney, Australia		13/05/2022

Chair, Executive Operations Secretariat, Australasian Kidney Trials Network

Name, Affiliation	Signature	Date
Prof Carmel Hawley<br>Australasian Kidney Trials Network, The University of Queensland, Brisbane, Australia		19/05/2202

This *Statistical Analysis Plan is* the property of the University of Queensland through the Australasian Kidney Trials Network. No section of this document may be copied, published, or used for any purpose without appropriate acknowledgement and the prior written permission of the Australasian Kidney Trials Network.

This Statistical Analysis Plan (SAP) describes the planned analyses and reporting for The Australasian Kidney Trials Network (AKTN) protocol 18.01, NAVKIDS^2 trial: A multicenter, waitlisted, randomised controlled trial of patient navigators in children with chronic kidney disease.

The structure and content of this SAP provide sufficient detail to meet the requirements identified by the International Council for Harmonisation of Technical Requirements for Registration of Pharmaceuticals for Human Use (ICH): Guidance on Statistical Principles in Clinical Trials (1). All work planned and reported for this SAP will follow national and international guidelines for statistical practice (1, 2).

The planned analyses identified in this SAP will be included in future manuscripts. Exploratory analyses not necessarily identified in this SAP may be performed to support planned analyses. Any post-hoc or unplanned analyses not specified in this SAP will be clearly identified as such in the Final Statistical Report (FSR) and manuscripts for publication.

This SAP was written and reviewed by the statisticians and clinical investigators from the NAVKIDS^2 Trial Steering Committee (TSC). All contributors were blinded to treatment allocations and treatment-related study results and will remain so until the central database is locked and the final data extracted for analysis.

The following documents were reviewed when preparing this SAP:

- Clinical Research Protocol for AKTN Trial Number 18.01 (3) and Published protocol (4)
- Electronic case report forms (eCRFs) and the Data Management Plan for AKTN Trial Number 18.01.
- Operations Manual (Version 4.0) for AKTN Trial Number 18.01
- Safety Monitoring Committee (SMC) Charter for AKTN Trial Number 18.01.
- ICH Harmonised Tripartite Guideline on Statistical Principles for Clinical Trials (1).
- ICH E9 (R1) Addendum on estimands and sensitivity analysis in clinical trials to the guideline on statistical principles for clinical trials (5).
- ICH Harmonised Tripartite Guideline on Structure and Content of Clinical Study Reports (6).

Readers of this SAP are encouraged to read the Clinical Research Protocol for further details on the conduct of this study and the operational aspects of clinical assessments and timing for completing a patient in this study.

ABBREVIATIONS

Abbreviation	Definition
AIHW	Australia Institute Health and Welfare
AKTN	Australasian Kidney Trials Network
ANZDATA	Australia and New Zealand Dialysis and Transplant
cLDA	Constrained longitudinal data analysis
CKD	Chronic kidney disease
CKD-D	Chronic kidney disease on dialysis
CKD-T	Chronic kidney disease with a transplant
CV	Cardiovascular
eGFR	Estimated glomerular filtration rate
eCRF	Electronic case report form
FDA	US Food and Drug Agency
FSR	Final statistical report
GEE	Generalised Estimating Equation
GLMM	Generalised Linear Mixed Model
HREC	Human Research Ethics Committee
HUI	Health Utility Index
ICH	International Council for Harmonisation of Technical Requirements for Registration of Pharmaceuticals for Human Use
iPTH	Intact parathyroid hormone
ITT	Intention-To-Treat
KCAD	Kids with CKD study
LMM	Linear mixed model
MAR	Missing at random
MBS	Medicare Benefits Schedule
MRFF	Medical Research Future Fund
NDI	National Death Index
OR	Odds ratio
PBS	Pharmaceutical Benefits Scheme
PedsQL	Pediatric Quality of Life Inventory™
PP	Per protocol
QoL	Quality of life
RA	Remoteness Areas

**REDCap**	Research Electronic Data Capture
**SAE**	Serious Adverse Event
**SAP**	Statistical Analysis Plan
**SD**	Standard deviation
**SES**	Socioeconomic status
**SRH**	Self-rated health
**SMC**	Safety Monitoring Committee
**TLF**	Tables, listings, figures
**TORCH**	Tools for Outcomes Research to measure and value Child Health (TORCH)
**TSC**	Trial Steering Committee

1. INTRODUCTION

1.1 Background

Chronic kidney disease (CKD) is a devastating illness associated with increased mortality, reduced quality of life, impaired growth, neurocognitive impairment and psychosocial maladjustment in children. The overall annual mortality rate for children on dialysis is 35 per 1000 population and is thirty-fold higher than children without CKD (7). Such large discrepancies in mortality rates remain unchanged despite medical advances over the past two decades.

The Kids with CKD (KCAD) study (8, 9) found that poor health in children with CKD is not only attributed to the direct influence of the chronic illness but also reflects outcomes of the complex pathway that defines equitable access to healthcare. The KCAD study found that children with CKD of the lowest and second lowest socioeconomic status (SES) quartiles were at least 3 and 2 times more likely to experience poorer overall health compared to the highest SES quartile (9). Previous work has also demonstrated the disparities in health among children with CKD are attributed to a myriad of different barriers including patient-level, health system and provider factors that extend beyond the biological differences (10). System level factors may include the inherent complexity of the health care system and poor access to primary or specialty care. At the provider level, barriers may include the lack of support systems to implement recommended care. Patient level barriers may include lack of awareness of resources, financial constraints, and competing priorities (e.g., family and work, and care for other siblings), that in turn make following complex care plans in the management of a child with CKD particularly challenging (11). Previous research has also indicated that children with kidney failure living in remote/regional Australia are less likely to access optimal care including pre-emptive living donor kidney transplantation (12). As such, there is a need to test novel interventions such as a community/patient navigator program that may improve such disparities in disadvantaged populations.

Patient navigators are trained non-medical personnel who assist patients with complex and/or chronic conditions journey through the continuum of care and transit across different care settings. They help the vulnerable and disadvantaged populations with chronic illness to better understand their diagnoses, treatment options, and available resources, to guide them through the complex medical system and to overcome barriers to health care access and bridge gaps in transitions of care (13). There is evidence supporting application of patient navigator programs in children with chronic conditions, such as diabetes, asthma and obesity, for improved access to care (14-16). Given the complexity and chronicity of the disease process and growing concerns that current models of care in paediatric nephrology may not be equipped to support the provision of high-level care to children with CKD from socio-economically disadvantaged backgrounds (9), the NAVKIDS^2 study hypothesises that a patient-navigation program will lead to improvement in the provision of care and overall health of children with CKD and is cost-effective.

1.2 Study synopsis

The NAVKIDS[2] trial is a multi-centre, waitlisted, randomised controlled trial that assesses the health benefits and costs of a patient navigator program in children with chronic kidney disease (CKD) stages 1-5, on dialysis (CKD-D) or with kidney transplants (CKD-T), who are disadvantaged with low socioeconomic backgrounds and/or living in rural/remote areas. The trial design and research plan are informed by the KCAD study (8). Eligible patients will be randomised to either immediately receive the patient navigator intervention for 6 months (intervention arm) or to be waitlisted with standard care until receipt of a patient navigator after 6 months (control arm). The planned recruitment target is 150-168 patients (further details are given below) across 5 sites.

The primary objective of the NAVKIDS[2] study is to compare the self-rated health (SRH) of children with CKD randomised to the immediate treatment (patient navigator program) arm and the waitlisted (control) arm. The secondary objectives are to compare other outcomes, including utility-based quality of life, caregiver SRH, caregiver's satisfaction with healthcare, progression of kidney dysfunction, other biomarkers, number of missed school days, number of hospitalisations, and mortality. The trial also includes an economic evaluation and process evaluation, which will assess the cost-effectiveness, fidelity and barriers and enablers of implementing a patient navigator program in this setting. Figure 1 displays the study schema.

1.3 Qualitative and Exploratory Studies

There are several exploratory and qualitative studies associated with the NAVKIDS[2] trial.

There will be a caregiver, patient navigator and healthcare professional substudy that consists of qualitative interviews from personnel from all sites.

The NAVKIDS[2] trial is also being conducted in collaboration with the MRFF Tools for Outcomes Research to measure and value Child Health (TORCH) project, in which exploratory measures of health-related quality of life will be included for a subset of trial participants. Data collected for these exploratory endpoints will be separately evaluated by the TORCH program team.

Data are also being collected on the primary and secondary outcomes up to 12 months post-randomisation and the waitlist group have additional measurements taken once they have begun the patient navigator program. A number of exploratory analyses will be performed using the data collected at these additional time points. These analyses may investigate the effect of waiting to receive the navigator intervention / address waitlist concerns and investigate long term follow-up for the immediate treatment arm to determine if the effect is sustained at 12 months post-randomisation. Data are also being collected on a number of biomarkers and exploratory analyses will investigate between group differences in the biomarkers.

Analyses for these exploratory studies will be documented separately.

Figure 1. Study schema

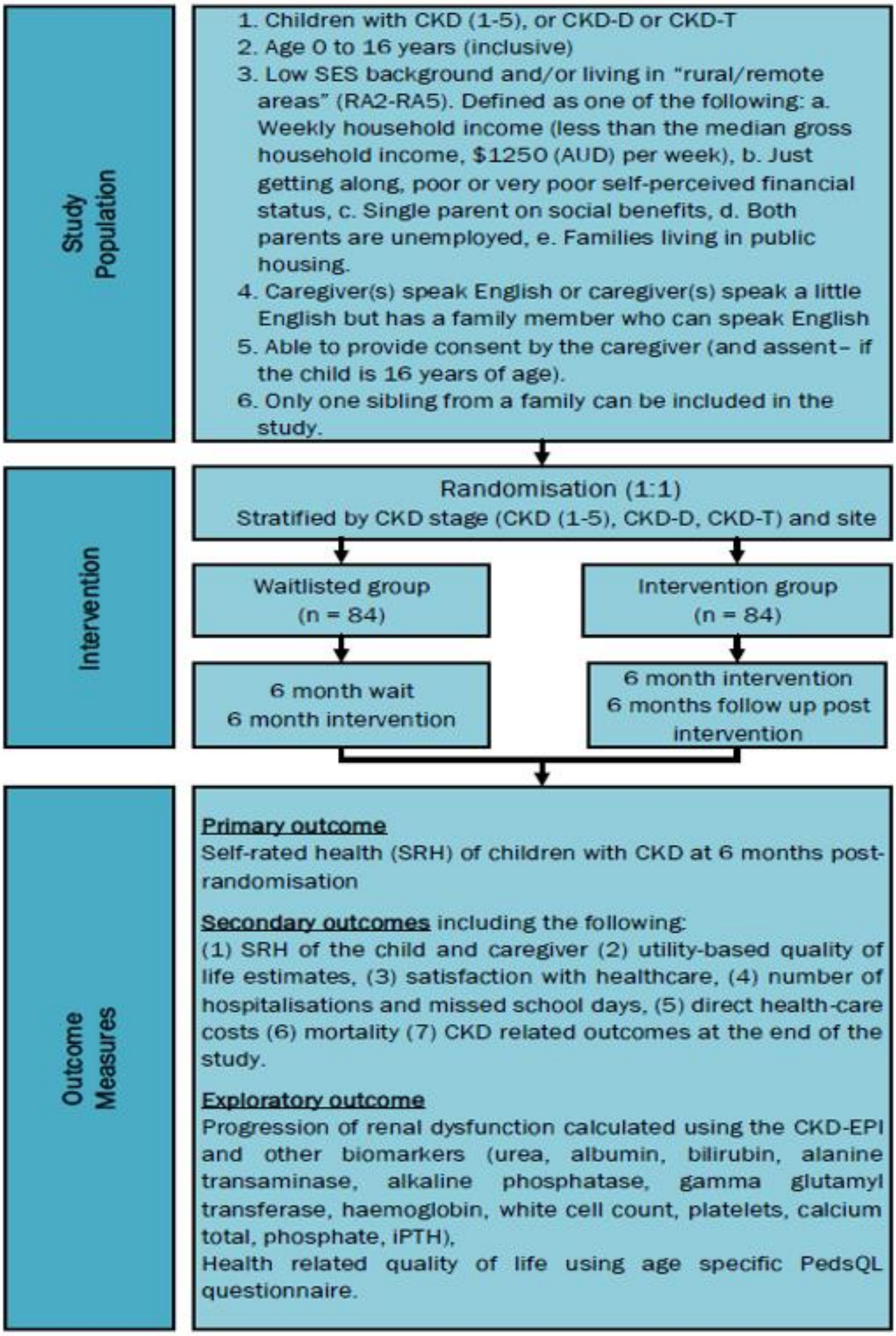

2. STUDY DESIGN

2.1 Overview

This study is a prospective, multi-centre, waitlisted, randomised controlled trial. NAVKIDS[2] is a superiority study and hypothesises that a patient-navigation program will lead to improvement in the provision of care and overall health of children with CKD and is cost-effective.

2.2 Study population

The population of interest is children (aged 0-16 years, inclusive) diagnosed with CKD 1-5, CKD-D or CKD-T that have a low SES background and/or are living in "rural/remote areas" (RA2-RA5) throughout Australia. Patients who met all inclusion and no exclusion criteria were considered eligible to participate in the trial.

2.2.1 Inclusion criteria

Patients were eligible for inclusion in the trial if <u>all</u> of the following criteria were met:

1. Children with CKD (1-5), or CKD-D or CKD-T.
2. Aged 0 -16 years (inclusive).
3. Low SES background and/or living in "rural/remote areas" (RA2-RA5). Low SES families are defined as one of the following (self-reported):
   a. Weekly household income (less than the median gross household income, $1250 (AUD) per week),
   b. Just getting along, poor or very poor self-perceived financial status,
   c. Single parenting on social benefits,
   d. Both parents are unemployed,
   e. Families living in public housing.
4. Caregiver(s) speak English or caregiver(s) speaks a little English but has a family member who can speak English
5. Able to provide consent by the caregiver (and assent– if the child is 16 years of age).
6. Only one sibling from a family can be included in the study

2.2.2 Exclusion criteria

Patients were excluded from the trial if the following condition was met:

1. Limited life expectancy of less than 12 months.

2.2.3 Protocol amendments to eligibility criteria

Several modifications were made to eligibility criteria after the trial commenced and the protocol paper was published (4). Below is a summary:

1. Changing inclusion criteria from 3-17 years to 0-16 years for version 5.0 of the protocol.

2. Inclusion criteria added: “Caregiver(s) speak English or caregiver(s) speaks a little English but has a family member who can speak English” for version 5.0 of the protocol.
3. Removed exclusion criteria and added inclusion criteria: exclusion criteria “Unable to provide consent by the caregiver (and assent – if the child is 16 years or over)” was removed and inclusion criteria “Able to provide consent by the caregiver (and assent – if the child is 16 years of age)” was added to version 5.0 of the protocol.
4. Changing inclusion criteria from CKD3-5 to CKD1-5 for version 6.0 of the protocol.
5. Changing inclusion criteria low SES definition for self-perceived financial status from “poor or very poor self-perceived financial status” to “Just getting along, poor or very poor self-perceived financial status” for version 6.0 of the protocol.
6. Changing inclusion criteria from “Low SES background” to expand to “Low SES background and/or living in ‘rural/remote areas’ (RA2-RA5)” for version 6.0 of the protocol.
7. Inclusion criteria added: “only one sibling from a family can be included in the study” was added to the list of inclusion criteria for version 8.0 of the protocol.

2.3 Study design and treatment allocation

A written informed consent will be obtained for the study. During the COVID-19 pandemic at times where written informed consent cannot be obtained, a verbal consent will be obtained.

Individuals who meet the inclusion criteria and have given informed written or verbal consent will be randomised with equal probability (1:1) to the immediate treatment (patient navigator) or the waitlisted (control) group, via an independent central web-based system. The randomisation sequence is generated by a computerised random number generator, using a random permuted block design with randomly chosen block sizes. Randomisation will be stratified by CKD stage (CKD (1-5), CKD-D, CKD-T) and site (5 sites). The permuted block will be used for each stratum (combination of CKD stage and site).

Children randomised to the immediate treatment arm will receive the intervention, the patient navigator program, immediately after randomisation for 6 months. Assessments (including the SRH of the child, utility-based quality of life, caregiver satisfaction with healthcare, number of missed school days) will be conducted pre-intervention, 1-month and 3-months into the intervention, immediately post-intervention and 6-months after the intervention.

Children randomised to the waitlist arm will wait for 6 months but receive the standard care during the ‘wait-period’ and commence the patient navigator program after 6 months. Assessments during the ‘wait-period’ will be conducted at baseline, 1-month and 3-months after randomisation and immediately pre-intervention, 1-month and 3-months into the intervention and immediately post-intervention. Similar to the immediate treatment arm, the waitlist-controlled arm will receive the patient navigator program for a period of 6 months.

The study design is summarised in Figure 2.

Figure 2 Study design diagram

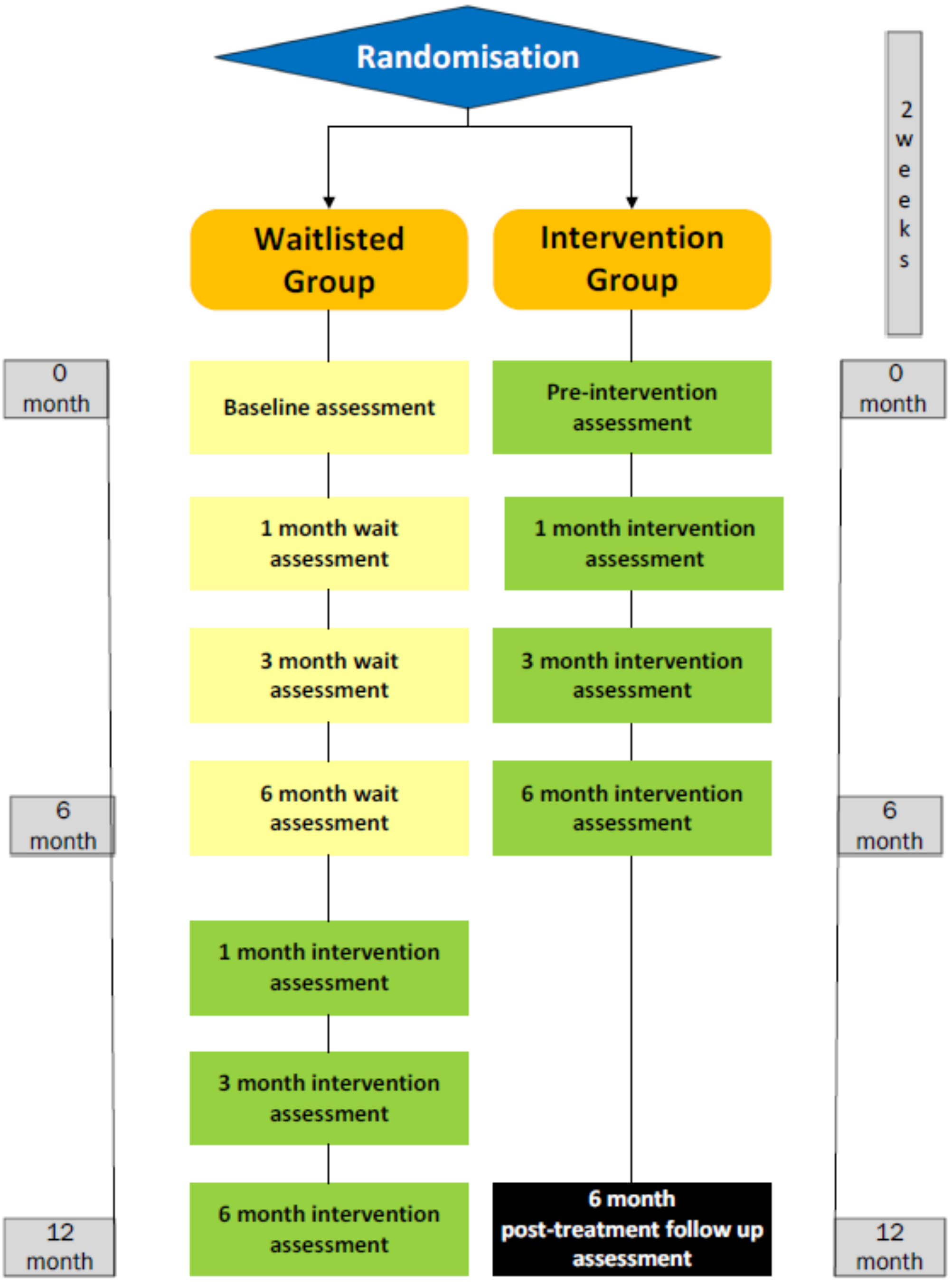

The primary paper will contain analyses performed on data collected prior to the waitlist group receiving the patient navigator intervention, i.e., data collected up until 6 months post-randomisation. Data collected after the waitlist group have begun the patient navigator intervention will be analysed in exploratory analyses that will be published in secondary papers.

2.4. Sample size

The sample size was calculated for the analysis of the 5-point Likert scale of the child SRH (and caregiver-rated health for younger children) using an ordinal logistic regression. Assuming an odds ratio (OR) of 2.3, we require approximately 168 patients to detect a significant effect at the 5% level with approximately 80% power. NAVKIDS[2] aims to recruit 150-168 patients as this is a practically feasible number for the study timeframe. It is assumed that the dropout rate will be low and so the sample size has not been inflated for dropout. Data from the KCAD study indicated the OR of children from the lowest SES quartile reporting poor and fair health (compared with good, very good and excellent health) was at least 2.0 (9). Therefore, an OR of 2.3 is a clinically significant change in SRH with the proposed intervention.

Table 1: Assumed distribution of the SRH (5-point Likert scale) for the control group (based on the KCAD study), and the power to detect the OR for the intervention (and the corresponding distribution of the outcome in the intervention arm). [The estimated change in the proportion of patients reporting good/very good and excellent health before and after the intervention is based on published estimates from a clinical trial of a complex intervention] (17).

OR	Power		Poor	Fair	Good	Very good	Excellent
		Intervention	4%	12%	50%	20%	14%
2.3	0.83	Control	9%	22%	51%	12%	7%

2.5 Treatment blinding and allocation concealment

The data analysts are blinded to treatment allocation. To ensure concealment of treatment allocation, randomisation is performed using an independent central web-based randomisation system administered by AKTN.

Blinding is not feasible for the patients, caregivers, clinicians or other researchers involved in study management.

2.6 Schedule of assessments

Table 2. Schedule of assessments for immediate treatment group

	Screen	Randomisation	Treat (1 month)	Treat (3 months)	End treat (6 months)	Post-treat (6 months)
**Visit Number**		1	2	3	4	5
**Day**		0 (Visit 1 within 2 weeks of rand)	28 +/- 2 weeks	84 +/- 2 weeks	168 +/- 2 weeks	336 +/- 2 weeks
**Eligibility Criteria**	x					
**Demographics**		x				
**CKD Information**		x	x	x	x	x
**Medical History**		x	x	x	x	x
**Physical Examination****		x	x	x	x	x
**Bloods****		x			x	x
**Immunosuppressive Medications**		x			x	
**Concomitant Medications**		x			x	
**SRH (Child and Caregiver)**		x	x	x	x	x
**Educational Background**		x				
**School Absenteeism**		x	x	x	x	x
**HUI Questionnaire***		x	x	x	x	x
**Caregiver Satisfaction Questionnaire (Caregiver)**		x	x	x	x	x
**Patient Navigator Satisfaction Questionnaire (Caregiver)**			x	x	x	
**Events of interest**			x	x	x	x
**Hospitalisation**			x	x	x	x
**Data linkage (NDI, ANZDATA, MBS, PBS)**						x
**Qualitative Interviews/Questionnaires (subset of participants only)**		x			x	
**PedsQL*****		x	x	x	x	x

mo = months
*HUI only to be completed by participants aged 3 years and above.
** Physical examination and blood test are not mandatory
***PedsQL completed only by some participants (TORCH study). PedsQL for ages 1 month and above.

Table 3. Schedule of assessments for waitlisted group

	Screen	Randomisation	Wait (1mo)	Wait (3mo)	Start treat	Treat (1mo)	Treat (3mo)	End treat (6mo)
Visit Number		1<br>(Visit 1 within 2 weeks of rand)	2<br>+/- 2 weeks	3<br>+/- 2 weeks	4<br>+/- 2 weeks	5<br>+/- 2 weeks	6<br>+/- 2 weeks	7<br>+/- 2 weeks
Day		0	28	84	168	196	252	336
Eligibility Criteria	x							
Demographics		x						
CKD Information		x	x	x	x	x	x	x
Medical History		x	x	x	x	x	x	x
Physical Examination**		x	x	x	x	x	x	x
Bloods**		x			x			x
Immunosuppressive Medications		x			x			x
Concomitant Medications		x			x			x
SRH (Child and Caregiver)		x	x	x	x	x	x	x
Educational Background		x						
School Absenteeism		x	x	x	x	x	x	x
HUI Questionnaire*		x	x	x	x	x	x	x
Caregiver Satisfaction Questionnaire (Caregiver)		x	x	x	x	x	x	x
Patient Navigator Satisfaction Questionnaire (Caregiver)						x	x	x
Events of interest			x	x	x	x	x	x
Hospitalisation			x	x	x	x	x	x
Data linkage (NDI, ANZDATA, MBS, PBS)								x
Qualitative interviews/questionnaires (subset of participants only)					x#			x
PedsQL***		x	x	x	x	x	x	x

mo = months
*HUI only to be completed by participants aged 3 years and above.
** Physical examination and blood test are not mandatory
# Waitlist group visit 4 interview can be done any time from post-randomisation to before the start of intervention
***PedsQL completed only by some participants (TORCH study). PedsQL for ages 1 month and above.

3. STUDY OUTCOME VARIABLES

3.1 Primary outcome

The primary outcome is the self-rated health (SRH) of children measured at 6 months post-randomisation. This is measured using a 5-point Likert scale. For children below the age of 8 (i.e., 0-7 years), caregiver-rated health of the child will be used as a proxy. For children that have not completed the SRH themselves (and are 8 years and above) but have a caregiver-rated health of the child completed, this will also be used as a proxy.

3.2 Secondary outcomes

Data are being collected on the following secondary outcomes up to 6 months post-randomisation:

1. SRH of the child over time (measured at baseline, 1 month, 3 months, and 6 months post-randomisation for both arms).
2. SRH of the caregiver (measured at baseline, 1 month, 3 months, and 6 months post-randomisation for both arms).
3. Utility-based quality of life (QoL), assessed using the Health Utility Index (HUI) for Children aged 3 and above (measured at baseline, 1 month, 3 months, and 6 months post-randomisation for both arms).
4. Caregivers' satisfaction with healthcare – assessed using the caregiver satisfaction questionnaire (measured at baseline, 1 month, 3 months, and 6 months post-randomisation for both arms).
5. All-cause hospitalisation (none, at least one) at 6 months post-randomisation for both arms.
6. All-cause hospitalisation (total number) at 6 months post-randomisation for both arms.
7. Number of missed school days (in the past 4 weeks; measured at baseline, 1 month, 3 months, and 6 months post-randomisation for both arms).

3.3 Exploratory outcomes

Data on the following biomarkers are being collected at baseline and 6 months post-randomisation and are viewed as exploratory outcomes:

1. Creatinine
2. Urea
3. Albumin
4. Bilirubin
5. Alanine transaminase
6. Alkaline phosphatase
7. Gamma glutamyl transferase
8. Calcium total
9. Phosphate
10. Intact parathyroid hormone
11. Haemoglobin
12. White cell count

13. Platelets

Progression of kidney dysfunction will be examined based on the estimated glomerular filtration rate (eGFR), calculated using a modified Schwartz equation, using serum creatinine collected at baseline and 6 months post-randomisation for both arms.

The Pediatric Quality of Life Inventory^{TM} (PedsQL) is being collected as an exploratory endpoint as part of the TORCH study (Section 1.3) and will not form part of the primary analyses of NAVKIDS[2].

3.4 Longer term follow up outcomes

Data are also being collected on the Secondary and Exploratory outcomes up to 12 months post-randomisation. The waitlist group also have additional measurements taken at 7 and 9 months post-randomisation for many of the Secondary Outcomes (1 and 3 months into receiving the patient navigator intervention). Supplemental exploratory analyses may be performed which compare the two arms from when the patients began the patient navigator intervention to treatment completion (after 6 months) to investigate the effect of waiting to receive the navigator intervention / address waitlist concerns. Exploratory analyses may also investigate long term follow-up for the immediate treatment arm to determine if the effect is sustained at 12 months post-randomisation. Pre and post treatment analyses may also be performed for the waitlisted arm.

Data on the following outcomes will also be obtained at the end of the study (secondary outcomes):

1. Direct healthcare costs – obtained using hospitalisations information (parent-reported and medical records) and linked Medicare Australia data for outpatient healthcare use (Pharmaceutical Benefit Scheme (PBS) and Medicare Benefits Schedule (MBS)). This will also include the costs of the patient navigator program. Healthcare cost outcomes will be calculated for the following time points: 3 months into treatment, immediately post-treatment in both groups and at 6 months post-treatment in the immediate treatment group.
2. Death from any cause (alive, dead), cardiovascular (CV) death, and non-CV related death at 12 months post-randomisation – obtained via data linkage with the national death index (NDI).
3. CKD-related outcomes among those on dialysis and with kidney transplants will be obtained via linkage (at 12 months, after completion of study) with the Australia and New Zealand Dialysis and Transplant (ANZDATA) registry

3.5 Safety outcomes

It is anticipated that significant risk/ events of interest related to the intervention and the study are very unlikely. The following safety variables are collected:

- Death from any cause, CV death, and non-CV related death at 12 months post-randomisation
- All-cause hospitalisation at 6 months post-randomisation
- Number of reports to the Safety Monitoring Committee (SMC) – child safety issues, safety of patient navigator, complaints.

3.6 Process evaluation

The following process evaluation measures will be collected and reported on in the Final Statistical Report (FSR):

- Patient navigator satisfaction questionnaire (collected at 1 and 3 months into treatment and immediately post-treatment)
- Patient navigator activity – completed by the patient navigator once a day covering all activities they completed for a patient

Other process evaluation measures will be collected using a mixed methods approach (see Protocol (3)).

4. SEQUENCE OF PLANNED ANALYSES

4.1 Interim analyses

There are no planned interim analyses for NAVKIDS^2 that will review unblinded data for the primary or secondary outcomes.

An independent SMC comprising of individuals with expertise in paediatrics, clinical trials, and clinical research in nephrology will safeguard the interests of study participants, families and the patient navigators. The committee's role is to provide independent and timely review of any safety issues that arise during and from the trial. Routinely collected trial SAEs will not be collected as hospitalisations are expected in this group due to CKD and other medical conditions. The TSC will retain sole decision-making responsibility for modifications to the trial.

4.2 Blinded reviews of primary outcome

A blinded review of the primary outcome data will be conducted to examine the distribution of the child SRH to determine the most appropriate model (e.g., binary or ordinal logistic regression). There are currently no other planned reviews of the primary outcome data, apart from those conducted prior to database lock (see below).

4.3 Final analyses and reporting

Planned analyses identified in the trial protocol and this SAP will be performed only after the last patient has completed the 12-month follow-up assessment visit, the REDCap™ database has been cleaned and locked, and results from the central laboratory, which will be received in Excel spreadsheets, have been cleaned and declared final. Blinded data review meetings will be held before locking the REDCap™ database and, again, before declaring final electronic copies of the data in Excel spreadsheets. There will be no un-blinded review and analyses will not commence until this SAP has been approved by the Lead Principal Investigators and Trial Statisticians and reviewed and approved by the TSC.

Key statistics and trial results from the final analyses will be presented to the TSC for discussion prior to completion of the FSR and subsequent manuscripts. Any post-hoc exploratory analyses performed to provide support for planned analyses but not identified in this SAP will be documented and reported in appendices to the FSR and clearly identified as unplanned analyses. All analyses and their interpretation will be conducted independently of the trial funders: the National Health and Medical Council Medical Research Future Fund Rare Cancers, Rare Diseases and Unmet Need Initiative.

A number of exploratory analyses will be performed on the NAVKIDS^2 dataset. These analyses may investigate the effect of waiting to receive the navigator intervention / address waitlist concerns, investigate long term followup up to 12 months post-randomisation and investigate between group differences in the biomarkers. These analyses will not be published in the primary trial paper and so the planned approach for their analysis will not be included in the SAP.

5. CHANGES TO STATISTICAL INFORMATION IN THE PROTOCOL

The statistical analysis methods in the protocol (v9.0) (3) correspond to those presented in the SAP.

6. ANALYSIS PRINCIPLES

6.1 Estimands and Analysis sets

6.1.1 Estimands

During the conduct of the trial there have been unplanned gaps in the delivery of the patient navigator intervention for some of the sites. These are intercurrent events caused by the absence of a patient navigator at a particular site.

The primary analyses will be conducted as close as possible to the intention-to-treat (ITT) ideal so that we may estimate the effect of the patient navigator as it would happen in real life (e.g., staff leaving, staff on holidays, families not engaging).

As a secondary analysis we are interested in estimating “the effect of the patient navigator intervention as used by the child’s family in a world where navigators would always be available.” Rather than using a strict intention-to-treat or “treatment policy” analysis (5) for this analysis, a “balanced estimand” (18) will be used which is the ITT effect that would have been observed if patients in both arms were affected by the intercurrent event. This proposed estimand is a variant of the “hypothetical estimand” discussed in the ICH E9 (R1) addendum (5). Calculation of this estimand is achieved by treating data that is collected for study participants at the affected site during the patient navigator’s absence as missing for both treatment arms (since this data does not contain information relevant to this estimand), to avoid confounding by centre. Participant data collected prior to the interruption of the patient navigator intervention delivery and after recommencement of the patient navigator intervention will be retained for these analyses. Since data for most outcomes are collected at multiple timepoints, these will be used to infer what might have been observed if the patient navigator were available (e.g., via likelihood-based repeated measures models).

Per protocol (PP) analyses may also be conducted as secondary analyses.

6.1.2 Analysis sets

For the primary outcome, all randomised participants with at least one post-randomisation SRH measurement (1 month, 3 months or 6 months), completed by either the child or caregiver, will be included in the "full analysis set" and analysed in the group to which they were randomly allocated, regardless of whether they received the assigned treatment and irrespective of any protocol deviations or violations.

The analysis set for secondary outcomes will be similarly defined. The analysis set for secondary outcomes with repeated measures will include those with at least one post-baseline measurement. The utility-based QoL (assessed using the HUI) is only collected for children aged 3 and above, and so analyses will be restricted to those that fall in this age group and have at least one post-baseline measurement. The analysis set for outcomes that are measured at a single time point (e.g., 6 months post-randomisation), such as all-cause hospitalisation, will be those with data collected at that timepoint.

If the patient navigator intervention is stopped permanently for any reason, the participant is to continue participating and data collection will continue until the final study follow-up time point. If a participant expresses a wish not to complete questionnaires, they will remain in the study and other trial-related data will be obtained from medical records unless the participant has explicitly stated they do not want any further information collected.

Missing values will be assumed to be missing at random (MAR). All study discontinuations will be captured in a figure illustrating participant progression through the trial.

6.2 Multicentre study and heterogeneity

Five centres will recruit approximately 28-42 participants each. Study centre will be included in the main analyses as a fixed effect. All sites are within Australia.

6.3 Multiple comparisons and multiplicity

There will be no adjustments for multiplicity, allowing reviewers to make their own adjustments to estimates if required. All statistical tests of significance will be two-sided and at the 5% level.

6.4 Covariate adjustment

The main statistical analyses of primary and secondary outcomes comparing the treatment arms will include CKD stage (CKD (1-5), CKD-D, CKD-T) and study centre as fixed effects. Additionally, outcomes with repeated measures will include the time point and the interaction between time point and treatment arm in the model. If the sample size allows, additional modelling (supplementary analyses) with *ad hoc* adjustments may be performed where baseline characteristics are not sufficiently balanced across the treatment groups.

6.5 Missing data

6.5.1 Missing outcome data

The proportions of participants missing each variable will be summarised in each arm and at each time point. Missing data from the database will be checked against the CONSORT diagram. The planned strategy for handling missing data for outcome variables will depend on the amount of missing data observed and the planned analyses for the outcomes.

For repeatedly measured outcomes, defined as outcomes with at least two post-baseline measurements, missing outcome data will be addressed using likelihood-based statistical models that allow inclusion of all randomised participants in analyses of the primary and secondary outcomes who have at least one post-randomisation outcome measure. For secondary outcomes that are measured at a single time point (e.g., all-cause hospitalisations), complete case analysis will be performed.

The utility-based QoL (assessed using the HUI) is only collected for children aged 3 and above, and so analyses will be restricted to those that fall in this age group.

6.5.2 Missing baseline covariate data

There will be no missing values on the stratification variables. Mean imputation will be used to replace missing values on other baseline variables used as covariates in supplementary covariate-adjusted analyses of the treatment effect. While mean imputation can bias statistical estimates in observational studies, this is not the case in randomised trials where randomisation ensures baseline variables are independent of treatment group (19, 20). Mean values will be calculated from the non-missing values for the baseline variable using pooled data from both treatment groups. For binary (coded 0 or 1) variables, the imputed mean will be rounded up to 1 or down to 0, whichever is nearest. Imputation will be performed using the median rather than the mean for skewed continuous variables that are not to be transformed. The number (percentage) of missing values will be reported for all baseline covariates with missing data.

7. STATISTICAL METHODS

The analyses outlined in this section will be conducted using the estimand framework described in Section 6.1.1. The significance level will be 5% (2-sided) for specified analyses. The estimated effect size and its precision (95% Confidence Intervals) will be presented for all primary and secondary outcomes (apart from those that have been pre-specified as descriptive analyses, e.g., mortality; see below).

7.1 Analysis of the primary outcome

The main statistical analyses are targeted at estimating the difference in the *SRH of the child* between participants randomised to the immediate treatment and waitlisted groups at 6 months post-randomisation. The estimates of such differences will address research objectives.

7.1.1 Primary comparison

Provided there are a sufficient number of participants with child SRH completed by the same person over time, we will analyse all repeated measures of child SRH (ordinal) from baseline and up to (inclusive) 6 months post-randomisation using a cumulative logit (proportional odds) mixed effects model (generalised linear mixed model (GLMM)). The model will include a random intercept for each participant and will examine interactions between treatment arm and time/visit (modelled as categorical). These models will be constrained (using a constrained longitudinal data analysis (cLDA) model) (21) so that a common baseline value is assumed and fit across the treatment arms. An unstructured variance-covariance matrix will be used and if the unstructured pattern fails to converge, more parsimonious error structures will be tested. These models will also be adjusted for the stratification factors: CKD stage (CKD (1-5), CKD-D, CKD-T) and site. The primary result will be the treatment effect estimate, presented as an OR, at 6 months post-randomisation and the 95% CI obtained from the model.

Prior to performing the ordinal logistic regression, the distribution of the child SRH will be checked to ensure that this model is appropriate. If, in the extreme case that the majority of the data falls into just two of the categories of the 5-item Likert scale, then binary logistic regressions will instead be implemented (GLMM). We may also consider collapsing the five categories into three or four categories and performing ordinal logistic regressions on the collapsed scale if some of the categories have low counts. The proportional odds assumption will be examined by performing separate logistic regressions to investigate how the ORs for the treatment effect vary at the different thresholds. If the proportional odds assumption is violated then partial proportional odds models may be used (generalised estimating equations (GEEs) may instead be required to fit these models). If model assumptions are unable to be satisfied, simple comparisons between the treatment arms will be performed.

7.1.2 Sensitivity to the person completing the SRH

The person completing the SRH of the child is recorded. A summary of the proportion of SRH completed by children and by caregivers will be provided, including breakdowns by age group (above 8 and below 8 years) for each treatment arm. Sensitivity analyses may be performed removing those that were caregiver-completed (for those over 8 years old). Consistency of the reporter for SRH of the child will also be examined across each time point; those with different people completing the SRH across the visits may also be removed in a sensitivity analysis.

7.1.3 Sensitivity to missing data mechanism

The analysis of the primary outcome assumes data are missing at random (MAR). While MAR is likely to be plausible for the majority of missing values, sensitivity to the MAR assumption may be assessed by analysing data with models that assume plausible MNAR assumptions.

7.2 Analysis of the secondary outcomes

SRH of the caregiver will be analysed using similar approaches to SRH of the child. Consistency of the person completing the SRH of the caregiver will also be checked.

Secondary endpoints that are continuous data, such as utility-based quality of life (HUI), will be analysed using linear mixed models to compare the immediate treatment and waitlisted groups at 6 months post-randomisation. These models will contain similar variables to the child SRH GLMMs.

Count data measured at a single time point, such as number of hospitalisations, will be analysed using Poisson regression (or negative binomial or zero-inflated Poisson regression if more appropriate) to compare the immediate treatment and waitlisted groups at 6 months post-randomisation, provided there are sufficient numbers. These models will include an offset variable to account for duration in the trial and will be adjusted for the stratification factors. These models will be extended to GLMMs for count outcomes measured at multiple time points, such as number of missed school days (in the past 4 weeks; no offset variable required). If the number of participants with hospitalisations or missed school days is small then descriptive analyses may be used in place of the above-mentioned analyses.

Binary outcomes, such as hospitalisation (none vs at least one) at 6 months post-randomisation, will be analysed using logistic regressions (adjusted for stratification factors) to compare the treatment arms, provided there are sufficient numbers of patients with (or without) hospitalisations. Descriptive analyses will be performed if the proportion of patients with hospitalisations is small (or if almost all patients have hospitalisations).

Descriptive analyses will be performed for the following outcomes:

- Death from any cause, CV death, and non-CV related death (expected to be low for this patient population)
- CKD-related outcomes (e.g., graft failure, rejection)
- Number of reports to the Safety Monitoring Committee (SMC) – child safety issues, safety of patient navigator
- Caregiver satisfaction questionnaire items at each time point

The patient navigator satisfaction questionnaires and patient navigator activity will be presented using descriptive statistics as part of the process evaluation.

The analyses of the exploratory outcomes will not be described here as they are secondary analyses.

A separation Health Economics Analysis Plan will be produced to describe the analysis plan for the direct health care costs.

7.3 Subgroup analyses

There are no planned subgroup analyses.

7.4 Sensitivity analyses

A sensitivity analysis of the HUI excluding children aged 3-4 will be performed. Whilst the HUI has been used for young children in previous studies, it has only been validated for children aged 5 and above, and so a sensitivity analysis will be conducted excluding children aged 3-4 years.

Depending on the proportions of caregivers completing the child SRH for those over the age of 8, sensitivity analyses may also be performed where the sample is restricted to child-completed SRH for those over the age of 8. Caregiver-completed child SRH for those under the age of 8 will remain in

the sample. Patients with inconsistent people completing the child and/or caregiver SRH across visits may also be removed for sensitivity analyses.

Several siblings have been recruited to the study (prior to a protocol amendment), some of whom have been randomised to different treatment arms. This will likely lead to contamination of those siblings randomised to the waitlisted group. As a sensitivity analysis we will re-run the primary analyses with these siblings removed.

As mentioned above, sensitivity analyses may be performed to investigate different missing data assumptions, and to investigate compliance with the study intervention (see below).

7.5 Impacts due to COVID-19

The COVID-19 pandemic has had and will continue to impact NAVKIDS[2]. Its effects on trial conduct and data may create multiple potential statistical issues.

Study recruitment/site initiation was delayed due to COVID-19. Due to budget concerns, follow-up was reduced from 24 months to 12 months post-randomisation (3, 4). This does not affect the primary analyses but affects secondary analyses which planned to investigate the long-term effects of the patient navigator intervention. During the COVID-19 pandemic outcome data may be missing for patients that are unable to attend some of their study visits. The trial may stop earlier than planned due to slow recruitment, which may result in loss of statistical power.

We will follow the guidelines provided by the US Food and Drug Agency (FDA) (22) and the Australian Government Department of Health (23) for statistical considerations for clinical trials during the COVID-19 public health emergency.

7.6 Treatment compliance (engagement with patient navigator)

Upon completion of the intervention the patient navigators will provide a summary of the level of engagement with the patient navigator program for each participant. This engagement will be rated as: Good (roughly 80% responsiveness; patient contactable and using patient navigator help), Reasonable (some willingness to converse/engage), or Poor (patient navigator not able to contact at all or family not engaging). Descriptive analyses will be performed to examine the level of engagement, the amount of contacts that the patients have with the patient navigators, and the types of activities that the patient navigators perform for the families.

Supplemental sensitivity analyses may be conducted (such as per protocol analyses) which account for the level of compliance/engagement with the patient navigator intervention.

7.7 Data manipulation and computing

All data manipulation, tables, figures, listings and analyses will be documented in SAS®, Stata® or R programs and performed using SAS version 9.4 or later, Stata version 17 or later, or R version 4.0.0 or later.

8. REPORTING

All results described above as well as tables, listings and figures (TLFs) listed below will be presented in the FSR.

8.1 Trial profile

All patients who provide informed consent will be accounted for in the FSR. A CONSORT-style flow diagram will illustrate patient progression through the trial from initial screening for eligibility to completion of the final outcome assessment (at 12 months post-randomisation). Number (percentage) of participants randomised to each treatment group will be given for all randomised patients along with reasons for study discontinuation (death, withdrawal of consent, at discretion of physician, lost to follow-up, transition to adult care, other) by treatment group. Reasons for study discontinuation will be reported at 6 months (relevant to primary outcome) and 12 months (final study visit) post-randomisation.

8.2 Protocol deviations

Protocol deviations falling within the following major categories will be reported: safety, informed consent, eligibility, protocol implementation, and other. Safety deviation sub-categories are: incident/hazard report was not done within 12 hours of incident/hazard detection, adverse event of interest was not reported to HREC, other. Informed consent deviation sub-categories are: failure to obtain informed consent, consent form used was not current HREC-approved version, consent form missing, missing signatures or dates on consent form prior to enrolment, other. Eligibility deviation sub-categories are: ineligible participant enrolled, randomisation error, other. Protocol implementation deviation sub-categories are: participant seen outside visit window, missed visit, assessment not done, assessment done outside window, other. The sub-categories for "other" protocol deviation will depend on the text responses. All protocol deviations will be reported by treatment group.

8.3 Patient characteristics and baseline comparisons

Demographic and other baseline characteristics will be summarised by assigned treatment group and overall. Categorical variables will be summarised by frequencies and percentages. Percentages will be calculated according to the number of patients for whom data are available. Where values are missing, the denominator, which will be less than the number of patients assigned to the treatment group, will be reported either in the body or a footnote in the summary table. Continuous variables will be summarised by mean and standard deviation (SD) as well as quartiles.

Variables to be included in the baseline table are: child age (years), sex, creatinine, eGFR, BMI, primary ethnicity (Middle Eastern, Anglo-Celtic, Asian, Aboriginal or Torres Strait Islander, Pacific Islander, Other), secondary ethnicity, languages spoken by child at home (English, other), Region (RA1-RA5; Remoteness Areas of Australia), marital status of primary caregiver(s) (single, partnered, married/defacto, divorced/separated, widowed), weekly household income ($0 - $599/week, $600 - $1,249/week, $1,250 - $1,999/week, > $2000/week; AUD), caregiver self-perception of family financial status (very poor, poor, just getting along, reasonably comfortable, very comfortable, prosperous), single caregiver on social benefits (yes, no), both caregivers unemployed (yes, no), family live in public housing (yes, no), comorbid conditions, primary causes of CKD (Glomerulonephritis/Glomerular disease/Vasculitis, structural and obstructive kidney disease, cystic kidney disease, unknown origin/other), CKD stage (CKD G1-5, CKD G5D, CKD G5T), previous

failed transplant (yes, no). Baseline characteristics of the primary and secondary/additional caregivers and summaries of immunosuppressive medication will also be provided.

8.4 Tables, Listings and Figures (TLFs)

8.4.1 Planned tables

The following are planned summary tables:

Table 1. Stratification variables by treatment group
Table 2. Demographic, medical history and clinical baseline characteristics by treatment group (table parts a, b, etc for main variables of interest and additional variables)
Table 3. CKD information by treatment group across study visits
Table 4. Clinical assessments by treatment group across study visits
Table 5. Withdrawals, protocol deviations and violations by treatment group
Table 6. Child and caregiver SRH by treatment group across study visits
Table 7. Estimates, confidence intervals and p-values from a generalised linear mixed model analysis of child SRH over time (table parts a, b, etc. for main, supporting, and sensitivity analyses)
Table 8. Estimates, confidence intervals and p-values from a generalised linear mixed model analysis of caregiver SRH over time (table parts a, b, etc. for main, supporting, and sensitivity analyses)
Table 9. HUI by treatment group across study visits
Table 10. Estimates, confidence intervals and p-values from a linear mixed model analysis of HUI over time (table parts a, b, etc. for main, supporting, and sensitivity analyses)
Table 11. All-cause hospitalisation (none, at least one) and number of all-cause hospitalisations at 6 months post-randomisation by treatment group
Table 12. Estimates, confidence intervals and p-values from logistic and Poisson/Negative Binomial regression analyses of all-cause hospitalisation (none, at least one) and number of all-cause hospitalisations
Table 13. Number of missed school days (for past 4 weeks) by treatment group across study visits
Table 14. Estimates, confidence intervals and p-values from a generalised linear mixed model analysis of number of missed school days over time
Table 15. Caregiver satisfaction questionnaire items by treatment group across study visits
Table 16. Patient navigator satisfaction questionnaires
Table 17. Patient navigator activity, patient navigator interruptions
Table 18. Treatment compliance for patients in immediate treatment arm; comparison of baseline variables by treatment compliance

8.4.2 Planned listings

The following are planned data and patient listings:

Listing 1. Patient deaths

Listing 2. All cause hospitalisations

Listing 3. CKD related outcomes

Listing 4. Reports to SMC – child safety issues and safety of patient navigator

Listing 5. Reasons for patients withdrawing from the intervention (permanent)

Listing 6. Reasons for temporary break from intervention

8.4.3 Planned figures

The following are planned summary figures:

Figure 1. Monthly and cumulative entry of participants into the study

Figure 2. Flowchart of patient progression through the study

Figure 3. Distribution and cumulative proportion of child SRH for each treatment arm at 6 months post-randomisation

Figure 4. Child SRH over time for each treatment arm

Figure 5 Marginal estimated probability plots of each category of child SRH over time for each treatment arm

Figure 6. Distribution and cumulative proportion of caregiver SRH for each treatment arm at 6 months post-randomisation

Figure 7. Caregiver SRH over time for each treatment arm

Figure 8 Marginal estimated probability plots of each category of caregiver SRH over time for each treatment arm

Figure 9. HUI over time

8.4.4 Supplementary TLFs

Results from supporting and sensitivity analyses will be presented in supplementary tables. Missing data on the primary outcome will be summarised by treatment groups and presented in supplementary tables and figures as appropriate.

8.5 General reporting conventions

All TLFs will be presented in portrait orientation, unless landscape orientation suggests that the information is easier to view. Legends will be used for all figures with more than one variable or item displayed. Figure lines should be wide enough to see the line after being copied.

All titles will be centred on a page. The first title line will be the number of the table, figure, or data listing. The second (and if required, third) line will be the description of the table, figure, or data listing. The ICH numbering convention will be used for all TLFs (6).

All tables, figures, and data listings will have the name of the relevant R/Stata/SAS program and a date-time stamp on the bottom of each output. All analysis programs developed for a table, figure, or data listing will be self-contained to facilitate transfer of programs to multiple computing environments. A separate analysis program will be written to produce each table.

8.6 Statistical summary conventions

For tables, sample sizes for each treatment group will be presented as totals in the column header (N=xxx), where appropriate. Sample sizes shown with summary statistics are the number (n) of patients with non-missing values.

Summaries for categorical variables will include only categories that patients had a response in. All summaries for continuous variables will include: N, mean, SD, and quartiles. Other summaries (e.g. 95% confidence intervals, coefficient of variation (CV) or %CV) will be used as appropriate. All percentages should be rounded and reported to a single decimal place (xx.x%). If percentages are reported as integers, percentages greater than 0% but <1% will be reported as <1%, whereas percentages greater than 99% but <100% will be reported as >99%. A percentage of 100% will be reported as 100%. Summaries that include p-values will report the p-value to three decimal places with a leading zero (0.001). P-values <0.001 will be reported as <0.001.

9. REFERENCES

1. ICH. ICH Harmonised Tripartite Guideline: Statistical Principles for Clinical Trials E9. 1998
2. ASA. Ethical guidelines for statistical practice. Prepared by the Committee on Professional Ethics.1999.
3. NAVKIDS2 Trial Protocol, Version 9.0, 06 December 2021
4. van Zwieten A, Caldwell P, Howard K, et al. NAV-KIDS2 trial: protocol for a multi-centre, staggered randomised controlled trial of a patient navigator intervention in children with chronic kidney disease. *BMC Nephrology*. 2019; 20:134.
5. ICH. ICH E9 (R1) Addendum on estimands and sensitivity analysis in clinical trials to the guideline on statistical principles for clinical trials. 2020.
6. ICH. ICH Harmonised Tripartite Guideline on Structure and Content of Clinical Study Reports E3. 1995
7. McDonald SP, Craig JC. Long-term survival of children with end-stage renal disease. *The New England journal of medicine.* 2004;350(26):2654-2662.
8. Wong G, Medway M, Didsbury M, et al. Health and wealth in children and adolescents with chronic kidney disease (K-CAD study). *BMC public health.* 2014;14:307
9. Didsbury M, van Zwieten A, Chen K, et al. The association between socioeconomic disadvantage and parent-rated health in children and adolescents with chronic kidney disease – the Kids with CKD (KCAD) study. *Pediatric Nephrol.* 2019; 34(7): 1237-1245.
10. Medway M, Tong A, Craig JC, et al. Parental perspectives on the financial impact of caring for a child with CKD. *American Journal of Kidney Diseases.* 2015;65(3):384-393.
11. Tong A, Sainsbury P, Chadban S, et al. Patients' experiences and perspectives of living with CKD. *American Journal of Kidney Diseases.* 2009;53(4):689-700.7.
12. Francis A, Didsbury M, Lim WH, et al. The impact of socioeconomic status and geographic remoteness on access to pre-emptive kidney transplantation and transplant outcomes among children. *Pediatr Nephrol.* 2016; 31:1011–1019.
13. Natale-Pereira A, Enard KR, Nevarez L, Jones LA. The role of patient navigators in eliminating health disparities. *Cancer.* 2011;117(15 Suppl):3543-3552
14. Van Walleghem N, Macdonald CA, Dean HJ. Evaluation of a systems navigator model for transition from pediatric to adult care for young adults with type 1 diabetes. *Diabetes care.* 2008;31(8):1529-1530.
15. Black HL, Priolo C, Akinyemi D, et al. Clearing clinical barriers: enhancing social support using a patient navigator for asthma care. *The Journal of asthma.* 2010;47(8):913-919.

16. Yun L, Boles RE, Haemer MA, et al. A randomized, home-based, childhood obesity intervention delivered by patient navigators. *BMC public health.* 2015;15:506
17. Bambauer KZ, Aupont O, Stone PH, et al. The effect of a telephone counseling intervention on self-rated health of cardiac patients. *Psychosomatic medicine.* 2005;67(4):539-545.
18. Michiels H, Sotto C, Vandebosch A, et al. A novel estimand to adjust for rescue treatment in randomized clinical trials. *Stat Med*. 2021; 40(9):2257-2271.
19. Groenwald RHH, White IR, Donders ART, et al. Missing covariate data in clinical research: when and when not to use the missing-indicator method for analysis. *Canadian Medical Association Journal.* 2012;184(11):1265-9.
20. Schemper M, Smith TL. Efficient evaluation of treatment effects in the presence of missing covariate values. *Statistics in Medicine*. 1990; 9:777-84.
21. Coffman CJ, Edelman D, Woolson RF. To condition or not condition? Analysing 'change' in longitudinal randomised controlled trials. *BMJ Open.* 2016; 6: e013096.
22. U.S. Food and Drug Administration: Statistical Considerations for Clinical Trials During the COVID-19 Public Health Emergency: Guidance for Industry. Food and Drug Administration, Rockville, MD, 2020. Available at: https://www.fda.gov/regulatory-information/search-fda-guidance-documents/statistical-considerations-clinical-trials-during-covid-19-public-health-emergency-guidance-industry. Accessed 27 October 2020.
23. Australian Government Department of Health. COVID-19: Guidance on clinical trials for institutions, HRECs, researchers and sponsors. 2020. Available at: https://www1.health.gov.au/internet/main/publishing.nsf/Content/EE207D978A44E4B8CA257FA90081B212/$File/CTPRG%20Statement%20on%20Clinical%20Trials%20during%20COVID-19.pdf Accessed 12/11/2020.
